# No difference in patient reported outcome and inflammatory response after coated and uncoated total knee arthroplasty – a randomized controlled study

**DOI:** 10.1186/s12891-023-07061-x

**Published:** 2023-12-14

**Authors:** Eric Tille, Franziska Beyer, Cornelia Lützner, Anne Postler, Peter Thomas, Burkhard Summer, Jörg Lützner

**Affiliations:** 1 University Center of Orthopaedic, Trauma and Plastic Surgery, University Hospital Carl Gustav Carus, TU Dresden, Fetscherst. 74, 01307 Dresden, Germany; 2 Department of Dermatology und Allergology, Ludwig-Maximilians-University, Munich, Germany

**Keywords:** Metal hypersensitivity, Total knee arthroplasty, Surface modification, Immunologic response

## Abstract

**Background:**

Allergies against implant materials are still not fully understood. Despite controversies about its relevance, some patients need treatment with hypoallergenic implants. This study compared coated and standard total knee arthroplasty (TKA) regarding inflammatory response and patient-reported outcome measures (PROMs).

**Methods:**

76 patients without self-reported allergies against implant materials were included in a RCT and received a coated or standard TKA of the same cemented posterior-stabilized knee system. 73 patients completed the 3-year follow-up. Two patients died and there was one revision surgery. Serum levels of cytokines with a possible role in implant allergy were measured in patient`s serum (IL-1beta, IL-5, IL-6, IL-8, IL-10, IFN γ, TNF α) prior to, one and three years after surgery. Furthermore, PROMs including knee function (Oxford Knee Score, Knee Society Score) and health-related quality of life (QoL, EuroQuol questionnaire) were assessed. Additionally, 8 patients with patch-test proven skin allergy against implant materials who received the coated implant were assessed similarly and compared to a matched-pair group receiving the same implant.

**Results:**

There were no differences in function and QoL between the assessed groups at any follow-up. The majority of patients demonstrated no elevation of the measured blood cytokines. Cytokine patterns showed no differences between study groups at any follow-up. The allergy patients demonstrated slower functional improvement and minor differences in cytokine pattern. Yet these results were not significant. There were no differences in the matched-pair analysis.

**Conclusion:**

We observed no relevant increase in serum cytokine levels in any group. The inflammatory response measured seems limited, even in allergy patients. Furthermore, there were no differences between coated and standard TKA in non-allergy patients in the 3-year Follow-Up period.

**Trial registration:**

The study protocol was registered in the US National Institutes of Health’s database (http://www.clinicaltrials.gov) registry under NCT03424174 on 03/17/2016.

## Background

Algorithms for the diagnostic approach of painful TKA have been introduced wherein hypersensitivity against implant materials is a possible cause of unexplained symptoms [1–3]. Yet, the relevance of allergies against implant materials is being discussed controversially [4–7]. There are reports of successful standard implants in patients with allergies, [8] but a growing number of reports has linked insufficient functional outcome, clinical symptoms and persistent pain to metal hypersensitivity [9–11].

Metal implants release ions due to wear and corrosion forming metallo-organic protein complexes which can be identified as agent by the immune system [12]. To address this issue hypoallergenic implants with surface modifications have been developed. Unfortunately, these implants show higher revision rates [13–16]. In a retrieval study hypoallergenic TKA demonstrated delamination potentially affecting the performance of the coating [14]. Therefore, a seven-layer zirconium nitride coating system (Advanced Surface - AS, B.Braun Aesculap, Tuttlingen, Germany) has been developed to improve coating quality [17]. This coating system demonstrated good long-term results [16, 18, 19].

Additionally several mediators have been reported to play a role in inflammatory reaction after TKA [20]. While some studies found increased IL-8 and IL-10 levels in standard TKA compared to coated TKA others did not find significant differences in blood cytokine patterns [21]. Cassuto et al. investigated inflammatory mediators, matrix proteins and bone regulating factors over a 20-year period after hip arthroplasty. He described response patterns and linked these to the phases of the healing process [22]. Generally, there is a lack of studies focusing on the inflammatory response after arthroplasty.

The underlying study was initiated to investigate the inflammatory response and patient-reported outcome measures (PROMs) in coated and standard TKA. It was hypothesized, that there would be a higher inflammatory response in standard TKA but no differences in PROMs compared to coated TKA.

## Methods

After institutional review board approval (IRB 00001473, IORG 00001076, registered at Office for Human Research Protection, EK 101,032,016) a randomized-controlled trial was conducted. The study protocol was registered prior to enrollment in the US National Institutes of Health’s database (http://www.clinicaltrials.gov) registry under NCT03424174 on 06/02/2018. The CONSORT reporting guidelines were used [23].

**Fig. 1:**
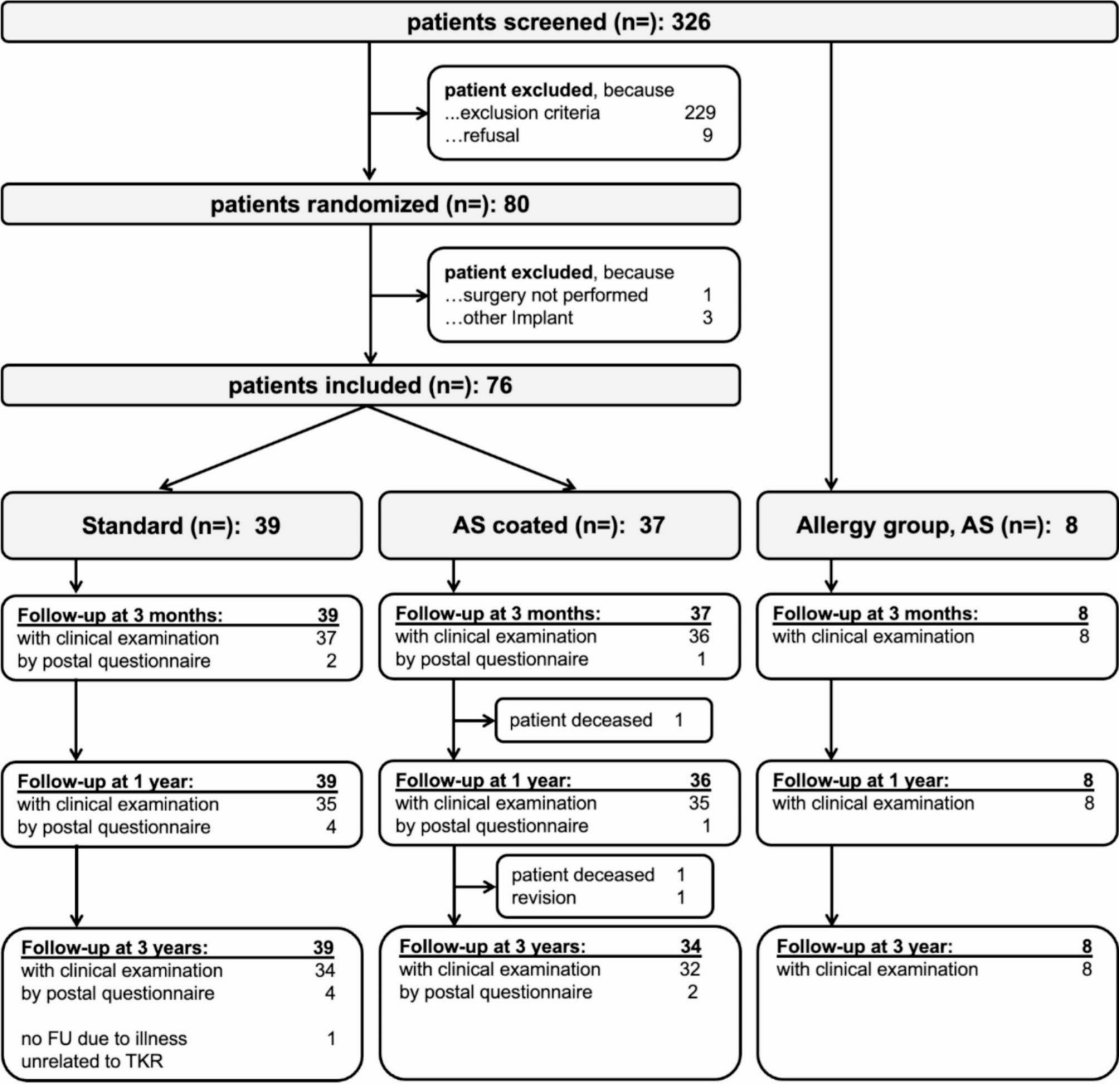
Flowchart of study patients enrollment and follow-up. *Blood cytokine pattern*

Patients scheduled for an unconstrained TKA without self-reported hypersensitivities against implant materials and without any existing metal implants were eligible to participate. After informed consent, a total of 80 patients were randomized using a software algorithm to receive a standard or coated TKA (Vega or Vega AS, B.Braun Aesculap, Tuttlingen, Germany). In one patient surgery was delayed due to medical problems and three patients needed a higher constraint during surgery. Therefore, a total of 76 patients was included in this study. An additional group including all patients with diagnosed allergies against one or more implant metals who presented during the study enrollment period was established (n = 8). Three patients of the allergy group were allergic to nickel only. Four patients had an allergy against nickel and cobalt and one patient presented with an allergy against nickel and palladium. These patients received the coated implant due to local guidelines (Fig. 1). In order to allow for comparability of the results we conducted a matched-pair analysis with non-allergic patients receiving a coated TKA. Matching criteria included age, sex, BMI and ASA-Score.

Both implants consisted of a CoCrMo-alloy (ISO 5832-4). The coated TKA had an additional multilayer coating system (Advanced Surface, AS) which was applied on the CoCrMo knee implants using a physical vapour deposition method in 7 layers with a gradient change in stiffness between the TKA body and the final layer with a total thickness of about 4 μm [19, 24]. All surgeries were performed by one of three experienced arthroplasty surgeons using a medial parapatellar approach and a tourniquet. All implants were posterior stabilized, cemented and no patellar resurfacing was performed.

Patients were seen by a study nurse prior to, 3 months, 1 year and 3 years after surgery. Knee Function (Oxford Knee Score, [OKS], Knee Society Score, [KSS]) and health-related quality of life (EQ-5D)) was assessed [25–28]. Furthermore, patients were asked about their overall satisfaction with the outcome of the TKA on a visual analogue scale (VAS) ranging from 0 (not satisfied) to 10 (very satisfied). 73 patients completed the 3year follow-up (FU) (Fig. 1).

Valid blood cytokine patterns were available from 74 patients from cryopreserved serum samples, which were stored at -20 °C. The blinded samples were assessed for the presence and concentration of 7cytokines by a multiplex cytometric bead assay (CBA; BD Biosciences, Heidelberg, Germany) via flow cytometry using a FACS canto (BD Biosciences, Heidelberg, Germany) [29]. The panel of cytokines included inflammatory (IL-1beta, IL-5, IL-6, IFN γ, TNF α), chemoattractant (IL-8,)) and immune regulating (IL-10) factors. The respective detection limit was < 0.01 pg/ml. The results were additionally evaluated by double assessment of 10 randomly selected blood samples.

### Statistical analysis

Sample size calculation was performed using data from a cross-sectional study with the same coating system [30]. To detect a difference of 1pg/ml in IL-8 or IL-10 between coated and standard implants with a power of 80% with a significance level of p < 0.05, a minimum of 31 patients per group were necessary. Accounting for loss-to-follow-up, 40 patients per group were included. Since most serum cytokine levels range between 5 and 40 pg/ml in healthy subjects the detection limit of 1pg/ml was chosen to ensure a sufficient level of sensitivity [31].

Data description was based on means and standard deviation (SD) for continuous values and absolute and relative frequencies for categorical values. Comparisons between treatment groups were done by Mann-Whitney-U-Test for continuous values and chi-square test for categorical values. Differences between normal and elevated cytokine levels with regard to PROMs were also analyzed by Mann-Whitney-U-Test. Significance level was set at p < 0.05. The software SPSS (release 26 for Windows) was used for data analysis.

## Results

**Table 1:** Baseline demographic characteristics of all patients who completed the 3-year Follow-Up given as mean with standard deviation or absolute (relative) frequency. Longer cut-sew time in allergy group due to suture instead of stapling. p-values: ^1^Non allergy patients receiving standard TKA vs. non-allergy patients receiving coated TKA. ^2^ Allergy group vs. coated non-allergy group (all patients). ^3^ Allergy group vs. matched-pair group. * = Chi2-Test, ‡ = Mann-Whitney-U-Test

	RCT				vs. Coated TKA		vs. allergy
	**Standard TKA** (n = 38)	**Coated TKA** (n = 34)	p-value^*1*^	**allergy** (n = 8)	p-value^2^	**Non-allergy coated (matched)** (n = 8)	p-value^3^
Age at surgery [years]	64.7 ± 9.6	63.8 ± 9.4	0.697	54.3 ±11.2	**0.018**	63.1 ±9.2	0.115*‡*
Female gender	19 (50%)	15 (44%)	0.618	8 (100%)	**0.036**	8 (100%)	n.a.
Comorbidities	22 (58%)	24 (71%)	0.263				
ASA grade 1 or 2	16 (42%)	10 (29%)		5 (63%)	0.575	5 (63%)	1.0*
ASA grade 3 or 4				3 (38%)		3 (38%)	
BMI [kg/m²]	30.6 ± 5.7	30.3 ± 5.9	0.858	33.9 ±6.7	0.054	30.0 ±4.9	0.141*‡*
cut-sew-time [minutes]	85.3 ± 15.9	89.1 ± 11.4	0.250	101.0 ± 8.7	**0.010**	81 ± 7	**0.002** *‡*
Surgeon	26 (68%)	24 (70%)	0.248				
Surgeon 1	9 (24%)	4 (12%)		6 (75%)		7 (88%)	
Surgeon 2	3 (8%)	6 (18%)		1 (12%)	0.725	1 (13%)	0.584*
Surgeon 3				1 (12%)		0 (0%)	

While the non-allergy groups were not different regarding pre- and perioperative data, such as gender, age, body mass index (BMI), surgery time and co-morbidities we found the allergy patients to be significantly younger. Moreover, the gender was not distributed equally in this group since all allergy patients were female. Also, the cut-sew time in this group was significantly longer. In order to address this issue and allow for comparability we established a matched-pair analysis (Table 1). One patient was not available for 3year FU due to medical reasons. Two patients in the coated group deceased as result of preexisting comorbidities. There was one revision after 2 years in the coated TKA group due to osteonecrosis of the medial tibial plateau and subsequent loosening of the tibial component. The revision was delayed due to further medical conditions and serious third body wear from bone cement occurred. Even in this catastrophic wear situation about 50% of the coating was still intact.

The blood cytokine patterns prior to surgery, one and three years after TKA demonstrated for most parameters no differences between the non-allergy groups (Table 2). Only for IFN γ there was a difference between the standard and coated groups at the 1year FU. However, this difference had already been present prior to surgery. At the 3year FU IFN γ levels had decreased and no differences were detected. In nearly all patients there were measurable levels of IL-8 (98%). IL-6 was measurable in 66% of the patients. Furthermore, few patients had measurable levels of IL-10 (12%), IL-5 (2%), IL1-ß (3%), IFN γ (2%) and TNF α (3%) at the 3year FU. Cytokine levels did not have an influence upon PROMs.

**Table 2:** Blood cytokine levels in Standard and Coated TKA (quantitative values given in pg/ml as median (quartiles), absolute (n) and relative (%) frequencies of values above 0.01 pg/ml). p-values: ^1^ Non allergy patients receiving standard TKA vs. non-allergy patients receiving coated TKA. ^2^ Allergy group vs. coated non-allergy group (all patients, Chi^2^-Test). ^3^ Allergy group vs. matched-pair group (Mann-Whitney U-Test)

	Standard TKA [n = 35]			Coated TKA [n = 37]				Allergy [n = 8]			vs. Coated TKA	Non-Allergy Matched [n = 8]			vs. Allergy
	median	n	%	median	n	%	**p-value** ^**1**^	median	n	%	**p-value** ^**2**^	median	n	%	**p-value** ^**3**^
**IL-1beta**															
prior to surgery	0 (0.0; 0.0)	4	11.4	0 (0.0; 0.0)	5	12.8	0.899	0 (0.0; 0.1)	2	25	0.493	0 (0.0; 0.0)	0	0	0.144
1 year follow-up	0 (0.0; 0.0)	2	5.7	0 (0.0; 0.0)	1	2.5	0.609	0 (0.0; 0.0)	0	0	0.717	0 (0.0; 0.0)	0	0	1.0
3-year follow-up	0 (0.0; 0.0)	1	3.1	0 (0.0; 0.0)	1	3.1	0.963	0 (0.0; 0.0)	0	0	0.894	0 (0.0; 0.0)	0	0	1.0
**IL-5**															
prior to surgery	0.1 (0.0; 0.4)	30	85.7	0.2 (0.0; 0.4)	29	74.4	0.427	0 (0.0; 0.1)	5	63	0.125	0.05 (0.01; 0.33)	6	75	0.455
1 year follow-up	0.1 (0.0; 0.2)	26	74.3	0.0 (0.1; 0.1)	28	71.8	0.824	0 (0.0; 0.1)	5	71	0.987	0.02 (0.0; 0.11)	4	50	0.905
3-year follow-up	0 (0.0; 0.0)	1	3.1	0 (0.0; 0.0)	0	0.0	0.329	0 (0.0; 0.0)	0	0	0.539	0 (0.0; 0.0)	0	0	1.0
**IL-6**															
prior to surgery	0 (0.0; 1.1)	15	42.9	0 (0.5; 0.5)	9	23.1	0.152	0 (0.0; 0.0)	0	0	**0.022**	0 (0.0; 0.0)	0	0	1.0
1 year follow-up	0.4 (0.0; 4.7)	18	51.4	1.8 (0.0; 4.9)	24	61.5	0.239	0 (0.0; 4.3)	3	43	0.401	1.7 (0.8; 3.5)	6	75	0.8
3-year follow-up	1.5 (0.0; 3.4)	22	68.8	0.7 (0.1; 1.4)	20	62.5	0.652	1.2 (0.0; 1.9)	5	71	0.829	0.9 (0.2; 1.3)	6	75	0.381
**IL-8**															
prior to surgery	6.2 (4.0; 9.2)	34	97.1	6.7 (3.3; 9.8)	38	97.4	0.952	6.0 (4.0; 29.6)	7	88	0.548	8.2 (5.8; 14.5)	8	100	0.674
1 year follow-up	3.3 (0.0; 6.1)	22	62.9	2.1 (0.0; 7.3)	20	51.3	0.420	0 (0.0; 2.8)	3	43	0.589	3.7 (0.0; 7.3)	5	63	0.180
3-year follow-up	5.4 (4.7; 9.0)	32	100	5.8 (4.5; 6.7)	31	96.9	0.298	4.6 (3.9; 6.4)	7	100	0.539	5.9 (5.3; 6.6)	8	100	0.325
**IL-10**															
prior to surgery	0.9 (0.8; 1.0)	35	100	0.9 (0.8; 1.1)	39	100	n.a.	1.1 (0.9; 1.2)	8	100	n.a.	0.8 (0.7; 0.9)	8	100	0.016
1 year follow-up	0.9 (0.6; 1.1)	35	100	0.8 (0.6; 1.0)	39	100	n.a.	0.8 (0.6; 1.2)	7	100	n.a.	0.7 (0.6; 0.8)	8	100	0.487
3-year follow-up	0 (0.0; 0.0)	5	15.6	0 (0.0; 0.0)	3	9.4	0.265	0 (0.0; 0.0)	0	0	0.447	0 (0.0; 0.0)	0	0	1.0
**IFN γ**															
prior to surgery	0 (0.0; 0.0)	3	8.6	0 (0.0; 0.0)	11	28.2	0.606	1.8 (1.3; 2.6)	8	100	**< 0.001**	0 (0.0; 0.0)	0	0	**< 0.001**
1 year follow-up	0 (0.0; 0.0)	9	25.7	0 (0.0; 0.2)	20	51.3	0.120	0.7 (0.1; 1.3)	6	86	**0.01**	0 (0.0; 0.1)	2	25	**0.038**
3-year follow-up	0 (0.0; 0.0)	0	0.0	0 (0.0; 0.0)	1	3.1	0.298	0 (0.0; 0.0)	0	0	0.539	0 (0.0; 0.0)	0	0	1.0
**TNF α**															
prior to surgery	0 (0.0; 0.0)	5	14.3	0 (0.0; 0.0)	6	15.4	0.825	0 (0.0; 0.1)	2	25	0.597	0 (0.0; 0.0)	0	0	0.144
1 year follow-up	0 (0.0; 0.0)	2	5.7	0 (0.0; 0.0)	1	2.6	0.609	0 (0.0; 0.0)	0	0	0.717	0 (0.0; 0.0)	0	0	1.0
3-year follow-up	0 (0.0; 0.0)	1	3.1	0 (0.0; 0.0)	1	3.1	0.963	0 (0.0; 0.0)	0	0	0.894	0 (0.0; 0.0)	0	0	1.0

In contrast to these findings, we observed no elevated IL-6 levels prior to surgery within the allergy group. This is significant compared to the non-allergy groups (p = 0.022). Within the matched-pair analysis, however, this was not evident. All allergy patients presented with measurable levels of IFN γ prior to surgery (p = < 0.001). IFN γ levels dropped at the 1year FU but were still significantly higher compared to the standard, coated and matched-pair group. Similarly, to the non-allergy cohorts IFN γ was not detectable at the 3year FU anymore. The further cytokines displayed no differences between the groups.

**Table 3:** PROMs of patients given as mean with standard deviation. p-values: ^1^ Non allergy patients receiving standard TKA vs. non-allergy patients receiving coated TKA. ^2^ Allergy group vs. coated non-allergy group (all patients, Chi^2^-Test). ^3^ Allergy group vs. matched-pair group (Mann-Whitney U-Test)

	RCT				vs. RCT		vs. allergy
	**Standard TKA** (n = 38)	**Coated TKA** (n = 34)	p-value^1^	**Allergy** (n = 8)	p-value^2^	**Non-Allergy matched** (n = 8)	p-value^3^
**Oxford Knee Score [0–48]**							
prior to surgery	20.7 ± 7.5	22.6 ± 7.4	0.296	23.3 ± 8.8	0.634	19.0 ± 7.6	0.293
1 year follow-up	38.5 ± 7.7	37.1 ± 6.7	0.417	27.5 ± 5.6	0.669	35.4 ± 6.3	0.635
3 year follow-up	38.5 ± 8.1	37.9 ± 8.0	0.748	36.1 ± 7.0	0.296	36.8 ± 8.4	0.223
**KSS Knee Score [0–100]**							
prior to surgery	40.0 ± 17.8	41.4 ± 17.7	0.742	42.3 ± 14.4	0.562	38.6 ± 16.5	0.563
1 year follow-up	88.9 ± 11.9	85.7 ± 12.9	0.294	76.9 ± 10.6	0.044	85.0 ± 13.7	0.187
3 year follow-up	86.5 ± 12.4	86.0 ± 14.8	0.868	91.1 ± 9.7	0.053	85.4 ± 14.7	0.751
**KSS Function Score [0–100]**							
prior to surgery	54.1 ± 17.2	56.8 ± 16.3	0.501	56.3 ± 19.2	0.855	50.6 ± 17.4	0.456
1 year follow-up	74.1 ± 19.1	83.8 ± 13.7	0.016*	75.0 ± 22.0	0.334	83.8 ± 15.1	0.420
3 year follow-up	71.7 ± 26.5	80.0 ± 21.6	0.154	82.5 ± 23.1	0.611	85.0 ± 14.1	0.914
**EuroQol Visual Analogue Scale [0–100]**							
prior to surgery	55.9 ± 18.2	52.9 ± 13.2	0.424	41.4 ± 15.4	0.038	42.5 ± 13.9	0.705
1 year follow-up	71.0 ± 19.1	78.4 ± 11.7	0.057	55.0 ± 21.2	0.405	80.0 ± 12.2	0.347
3 year follow-up	69.5 ± 21.7	75.2 ± 16.6	0.214	74.6 ± 11.8	0.044	79.4 ± 14.0	0.048
**Subjective Satisfaction [0–10]**							
1 year follow-up	8.5 ± 1.7	8.3 ± 2.0	0.664	8.3 ± 1.5	0.565	8.1 ± 2.7	0.667

Within the non-allergy groups, we found no relevant differences in PROMs and ROM at any FU (Table 3). At the 1year FU the KSS Function score was significantly better in the coated group (p = 0.016). This resolved at the 3year FU.

Compared to these groups we observed a slower improvement within the allergy cohort. At the 1year FU the patient-reported scores in the allergy group were lower compared to the non-allergy groups. Yet these lower results were not significant.

Satisfaction was high in all groups, with a mean of 8.3 (± 2.0) in coated, 8.5 (± 1.7) in standard, 8.3 (± 1.5) in the allergy and 8.1 ± 2.7 in the matched-pair cohort.

## Discussion

This study demonstrated no relevant differences in inflammatory response, cytokine expression patterns and PROMs between coated and standard TKA in patients without allergies during mid-term FU.

Regarding the inflammatory response there were no differences between the standard and coated treatment group. In comparison to the allergy cohort, we found differences especially for the expression of IL-6 and IFN γ.

IL-5, a messenger for type-1 allergic reactions was detectable prior to surgery as well as at the 1year FU. This was unexpected since IL-5 is involved in acute reactions of the immune system modulating the change of antibody class in lymphocytes to IgE causing mast cell degranulation. This however is not a mechanism for metal hypersensitivity [4]. The measurable serum levels of IL-5 remain therefore not fully understood but resolved at the 3year FU.

IL-6 is a cytokine involved in the regulation of acute-phase proteins and immune response towards acute inflammation [32]. We detected increased IL-6 levels for standard and coated TKA patients at all timepoints. Interestingly within the allergy group and similar non-allergy patients (matched pairs) IL-6 was not detectable prior to surgery. The rise of IL-6 may be a hint towards a local tissue reaction or chronic inflammatory process after TKA. Previous studies have suggested that IL-6 production could also be an indicator for periprosthetic joint infection (PJI) [33]. However, with more than 60% of the patients producing measurable Il-6 levels and no PJI diagnosed, this association seems – at least for the underlying study - unlikely. Other authors suggested that high preoperative levels of IL-6 might be associated with pain severity or ongoing osteoarthritis while persisting high levels of IL-6 after TKA might be a predictor of insufficient pain relief [34, 35].

IL-8 is a proinflammatory cytokine which induces chemotaxis in neutrophils and other granulocytes causing migration to the site of immune reaction [36]. We observed measurable levels for most patients prior to surgery. This could be a sign of chronic osteoarthritis and its local inflammation [37]. After TKA the levels of IL-8 decreased by about 1/3 in both non-allergy groups suggesting a reduction of the inflammatory process. However, at the 3year FU IL-8 was detectable again in most patients. Since IL-8 can be triggered by various conditions (i.e. liver fibrosis) there might be no connection to the implanted prosthesis [38, 39]. On the other hand, this could be a sign of an immune reaction in which the body is dealing with the implant materials as other authors have shown an association between IL-8 expression and metal exposure [40, 41]. Thomas et al. showed an increase of IL-8 after standard TKA compared to coated TKA while Lützner et al. showed a correlation between worse functional outcome and elevated IL-8 levels [30, 42]. This, however, does not match our results. The reasons for the different results remain unclear. Yet there are studies suggesting a timely fluctuation of cytokine levels in the peripheral blood [22].

IFN γ has multiple functions. Among these, it works as a proinflammatory cytokine modulating the immune system during infection [43]. While we found no differences between standard and coated TKA, the IFN γ levels in the allergy group were significantly higher at the 1year FU. This was also true comparing the allergy patients with non-allergy matched pairs. This may suggest an ongoing effort of the body adapting to the implant material before reaching a tolerance. However, there might be a relevant bias, since IFN γ levels were elevated already prior to surgical treatment. At the 3year FU IFN γ had decreased in all cohorts.

IL-10 has a regulatory function. It acts anti-inflammatory and inhibits the expression of other cytokines. Within our study we found measurable levels of IL-10 prior to and during the 1year FU in every patient. At the 3year FU only few patients presented with measurable blood levels. A possible function might be to counteract the inflammatory processes mediated by IL-8 and IFN γ as discussed above. The elevated levels of IL-8 and IL-10 are in contrast to previously published results [42]. The specific reasons for elevation remain unclear.

The allergy group demonstrated several interesting findings. All patients were allergic to nickel and four concomitantly to cobalt. Metal allergy as delayed type hypersensitivity (DTH) is characterized by predominant Th1-type inflammation with IFN γ being a marker-signaling factor. Correspondingly, detectable IFN γ in blood characterizes the “DTH favouring status” – being visible already in the preoperative blood samples. What affects IFN γ in the serum to finally drop under the detection limit at the 3year FU is difficult to decipher. A transition to counteracting cytokine patterns – at least to Th2 or immune dampening - is not evident from our data, since levels of IL-5 are unchanged and IL-10 is even decreasing. Are (internal) sensitizing allergen and particle contacts decreased thus reducing the generation of new Th1 effector cells? This constellation may be given using surface coated implants and by the disappearance of initial periimplant saw blade/cutting guide derived particles [44, 45]. Other factors such as reduced external contact to problem eliciting nickel containing items by the patients or further yet unknown anti-inflammatory properties of surface coated implants may contribute.

The elevated serum levels for IL-5, -6, -8 and -10 prior to surgery might resemble the ongoing inflammatory reactions of the underlying osteoarthritis [37]. However, there is also the possibility that the elevation is due to confounding factors (i.e. comorbidities, smoking, etc.). Further cytokines such as IL1-ß and TNF-α presented without relevant measurable changes. The serum cytokine levels did not seem to have an effect upon the PROMs evaluated. Possible confounding factors biasing the blood testing were addressed by standardized storage of the samples and temporary exclusion of patients during periods of minor infections or other invasive measures (i.e. dental treatment). Furthermore, repeated controls yielded similar results accounting for a valid examination technique.

While hypersensitivities against implant materials have been known for a long time there is an ongoing debate about its importance in TKA. Especially, whether or not hypoallergenic implants are necessary is being discussed controversially [4–7, 46]. Some authors state that there is no evidence for the use of hypoallergenic implants [5, 8]. Others advise the use for patients with self-reported history of metal hypersensitivity [47–49]. Within these patients skin patch testing is the widely accepted diagnostic standard [50, 51]. This is despite the fact, that skin testing can – depending upon the evaluated substance – have a lower sensitivity than the more complex lymphocyte transformation test [LTT] [52, 53]. Yet both tests might not lead to a conclusion regarding joint tissue reaction [4, 54]. While the reported prevalence for metal hypersensitivity using skin patch testing is up to 32% [55], a recent study using LTT revealed a prevalence of only 3% [54]. Due to the limited specificity of the tests, general assessment of TKA patients without self-reported allergies is not recommended.

Regardless of these debates, there are patients with a diagnosed metal allergy asking for hypoallergenic implants. Guidelines and legal regulations differ between countries, making either the use of hypoallergenic implants or extensive informed consent a requirement to use standard implants. It is known that these patients are at higher risk for early revision and less satisfactory results. Therefore, refusing them a hypoallergenic implant is often difficult [16, 52, 54, 56, 57].

Even though coated implants have proven a better resistance against wear in vitro and the AS coating used in this study has demonstrated excellent long-term results, data of arthroplasty registries have suggested higher overall revision rates for hypoallergenic implants [17, 58, 59]. It needs to be considered that these implants are being used in patients with diagnosed or presumed hypersensitivity against implant materials. The coating itself is therefore very likely not the primary reason leading to revision. Generally, patients with metal hypersensitivity have shown worse outcome in TKA and total hip arthroplasty regardless of the implant used [56, 57, 60, 61]. The reasons are not fully understood. A connection between allergies, depression and anxiety disorders has been suggested since patients with a high level of psychological distress and anxiety tend to evaluate the outcome of TKA worse [62–64]. This occurs, even though the self-reported improvement after TKA (delta) is similar to psychologically healthy patients [62]. Since women are affected more often by anxiety disorders and allergies alike, this might explain for at least a certain percentage of differences [65–68]. Further reasons for the worse outcome need to be elucidated. In order to compare homogenous patient collectives and due to local guidelines, in the present study patients with self-reported metal allergy were excluded from randomization and observed as an additional group. This allows to compare the results of the standard implants directly to the coated implants eliminating biasing factors due to patient-specific characteristics. Regarding demographic parameters we found no differences between the standard and coated group. The allergy patients however were significantly younger. This may be a hint towards an earlier pain onset. Furthermore, all patients of this group were female. This is in line with the aforementioned higher percentage of women being affected by metal hypersensitivities. In order to address this demographic imbalance, we conducted a matched-pair analysis. Comparing the allergy cohort to similar non-allergy patients we found no differences in knee function and PROMs. This is consistent with previous findings in literature. While there are only few studies comparing coated to standard TKA, all of these demonstrated similar results [15, 69–73]. Comparing coated TKA to the allergy cohort we found a trend towards a slower rehabilitation within the allergy patients. While they reached similar scores at the 3year FU it seemed as though they needed longer. Yet these results were not significant and can therefore only be described as a trend. The reasons for this trend remain unclear.

To our knowledge, the present study is the first randomized controlled trial reporting longitudinal cytokine patterns after coated and standard TKA. It has, however, some limitations. This is mainly the exclusion of patients with a history of metal allergy and therefore the target population for coated implants. As explained, this was inevitable according to local guidelines. Yet, the allergy group demonstrated similar results, although this group included only a limited number of patients. Excluding patients with any kind of metal implants was necessary to avoid bias through these implants. This may have resulted in a study population with lower musculoskeletal comorbidities, potentially influencing the PROMs. Furthermore, a 3year FU is relatively short for patients following TKA. There might be changes between the groups at a later stage. Another limitation concerns the intake of anti-inflammatory drugs which may influence the serum cytokine levels. The duration and total amount of NSAR intake, however, was not evaluated during the FU. Also, it needs to be mentioned that the expression patterns of cytokines and other inflammatory mediators are fluctuating and not fully understood.

## Conclusion

There were no differences in blood cytokine patterns and PROMs between standard and coated TKA during mid-term follow up. The inflammatory response measured with several cytokines was low in all assessed groups. The use of the investigated coating system did not lead to higher complication rates or a worse outcome. It therefore seems to be a safe treatment option in patients who need a hypoallergenic implant.

## Data Availability

The datasets used and/or analysed during the current study are available from the corresponding author on reasonable request.

## Abbreviations

BMI: Body mass index
CoCrMo: Cobalt chrome mylobdenum
DTH: Delayed type hypersensitivity
EQ-5D: European Quality of Life 5 Dimensions 3 Level Version
FACS: Fluorescence activated cell sorting
FU: Follow up
IFN: Interferon
IgE: Immunoglobulin E
IL: Interleukin
KSS: Knee Society Score
LTT: Lymphocyte transformation test
OKS: Oxford Knee Score
PJI: Periprosthetic joint infection
PROM: Patient reported outcome
ROM: Range of motion
RT: Randomized trial
SD: Standard deviation
Th: T-helper (cells)
TKA: Total knee arthroplasty
VAS: Visual analogue scale
